# Histopathologic responses of the dental pulp to an experimental calcium aluminate–calcium silicate based capping material in comparison to mineral trioxide aggregate

**DOI:** 10.1038/s41598-026-41284-x

**Published:** 2026-03-21

**Authors:** Reham S. Saleh, Eyad A. Elbattawy, Shymaa Hamza, Yousra Aly

**Affiliations:** 1 Restorative and Dental Materials Department, National Research Centre, Cairo, Egypt; 2 Oral and Maxillofacial Pathology Department, Faculty of Dentistry, Cairo University, Cairo, Egypt

**Keywords:** Calcium aluminate, Calcium silicate, Pulp capping, Inflammation, Dentin bridge, Health care, Materials science, Medical research

## Abstract

The aim of this study was to compare the effect of Calcium Aluminate- Calcium Silicate based experimental pulp capping material with the commercially available Mineral Trioxide Aggregate (MTA) in terms of dentin bridge formation and pulp response. Four healthy male dogs were selected for this study. Teeth were randomly divided into four main groups (n = 14). Group I: experimental Calcium aluminate pulp capping material was used for pulp capping, Group II: Mineral Trioxide Aggregate pulp capping material was used for pulp capping, Group III: positive control group; no pulp capping material was applied and Group IV: negative control group; intact teeth were selected**.** Inflammatory response and dentin bridge formation was assessed at 1 month and 3 months. Regarding the inflammatory response; after 1 month, there was a statistically significant difference among all groups with *P* value < 0.001. Where MTA group showed a statistically significant lower inflammatory scores than the experimental Calcium aluminate group. After 3 months, there was a statistically significant difference among all groups with *P* value < 0.001. Where there was no statistically significant difference in the inflammatory scores between MTA and the experimental Calcium aluminate groups. For dentin bridge formation; at 3 months, there was a statistically significant difference among all groups at *p* value < 0.001. Where there was no statistically significant difference between MTA and the experimental Calcium aluminate groups. The new experimental Calcium aluminate-based biomaterial demonstrated good reparative capabilities and achieved the status of a potential material for use in vital pulp treatment.

## Introduction

Nowadays, there is a great demand towards the shift to the more conservative and minimally invasive approaches with preservation of the tooth vitality. Pulp exposure can occur due to carious insult, traumatic injuries or iatrogenic causes which may result in pulpal inflammation and infection as well. Maintaining pulp vitality is considered the main goal of vital pulp therapy which aim to prevent further complications that can lead to more radicular and invasive treatment approach^1^. Direct pulp capping DPC is the most suitable noninvasive vital pulp therapy in such cases with certain circumstances to grant its success^1^.

Several materials have been introduced in the dental market for direct pulp capping therapy, calcium hydroxide either chemically or light cured was commonly used for a long period of time as a gold standard material for pulp capping procedure^2^. The declaration of need an ideal pulp capping material was not only due to its drawbacks; such as high solubility, poor physico-mechanical properties and lack of adhesion, but also due to the creation of discontinuities in the formed dentin bridge and the possibility of obliterating the pulp chamber as well^3,4^.

Since 1990, Torabinejad and his coworkers had introduced mineral trioxide aggregate MTA as biocompatible root canal repair material as well as pulp capping material. In addition to its desirable physico-mechanical properties and lack of microleakage, it promotes regeneration of a good quality dentin bridge which directly influence the treatment prognosis in addition to its favorable pulp response^5^. On the other hand, MTA suffers from some drawbacks like; long setting time, difficult handling, poor mechanical properties and high cost^5^.

Recently, Calcium aluminate-based material acted as one of the promising bio-inductive alternative to MTA, due to its favorable physicochemical characteristics which resemble the tooth hard tissues. The particular formulation of the experimental material used in this study was based on the fact that, it is a Calcium aluminate- Calcium silicate-based material with Calcium carbonate and mixed with polyacrylic acid liquid. Calcium aluminate is characterized by having high mechanical properties^6^, favorable setting time and a rapid hydration rate, with capability to act as a barrier to prevent bacterial microleakage. Moreover, it is capable to induce in situ hydroxyapatite since its chemical composition and thermal expansion coefficient are similar to those of teeth and human bones^7^. In addition, it has the ability to form a layer of a biologically active apatite as soon as it exposed to anybody fluid and this property permit calcium release with the raise of its pH to allow cellular stimulation reparative dentin formation with an appropriate beneficial effect on the pulp tissue^8^. When used in pulpotomy procedures on rat teeth, Calcium aluminates showed a dentinogenic capability comparable to that of WMTA^9^. Calcium aluminate cement succeeded to show a comparable effect to Biodentine regarding pulp inflammatory response and dentin bridge formation in a recent study conducted on rats^10^. Similarly, in an experiment on Sprague–Dawley rats, Kramer et al.^9^ proved a dentinogenic capability of Calcium aluminate cement (Quick-Set) and Calcium silicate cements (ProRoot MTA and MTA Plus). After 30 and 60 days, all treated teeth maintained vitality and dentin bridge formation was observed with all three cements.

While the addition of tricalcium silicate C_3_S to tricalcium aluminate improved the compressive strength, reduced the setting time and helped in acceleration of the hydration process^11^. Moreover, ElBatanony et al.^12^ found that blending C_2_S into C_3_S improves the mechanical strength, and the handling properties of the produced cements and a reliable setting time was obtained. Besides, they proved that this combination can help in proliferation and differentiation of human Dental Pulp stem cells (hDPSC)^12^ Calcium silicate-based materials are well known by their stimulation of biological repair. This is in accordance with the concept supporting the trend of developing materials with favorable sealing and biological properties^13^.

Adding Calcium carbonate CaCO_3_ to silicate cements, helps in attaining early strength, may also positively influence the bioactivity of silicate cements and improving its overall performance^14^.

In our study, dogs were selected to observe the effects of our smart innovative pulp capping material on vital pulp tissues, as they are similar to humans in the dentinogenesis mechanism with shorter period of time^15^.

Hence, it was of interest to formulate a new smart bio inductive based pulp capping material that not only induce a formation of high-quality reparative dentin bridge but also attain a favorable pulp response. Moreover, this study was conducted to compare the effect of Calcium aluminate- Calcium silicate based experimental pulp capping material with the commercially available MTA Angelus in terms of pulp response and dentin bridge.

Thus, the null hypothesis assumed that calcium aluminate-based pulp capping material can’t reduce pulpal inflammation nor induce reparative dentin in comparison to MTA.

## Materials and methods

### Materials

1. Two different pulp capping materials were used in our study; Mineral Trioxide Aggregate (MTA Angelus) and an experimental Calcium aluminate–Calcium silicate-based paste. (As shown in Table 1)
2. Hybrid Self-Cured Glass Ionomer (Equia fil GIC). (As shown in Table 1)

**Table 1:** Materials name, composition, brand name and manufactures.

Materials	Composition	Brand name	Manufactures
MTA Angelus	Powder: mixture of tricalcium silicate C_3_S, dicalcium silicate C_2_S, tricalcium aluminate C_3_A, Bismuth oxide Bi_2_O_3_ Liquid: distilled water	MTA angelus	Londrina-PR-Brazil
Experimental	Powder: mixture of 40% Calcium aluminate, 30% C_3_S, 10% C_2_S, 10% Calcium Carbonate (CaCo) and 10% Bismuth oxide Liquid: polyacrylic acid	Experimental	Powder is manufactured in National Research Centre Lab Liquid is from 3M,ESP, USA
Hybrid self-cured glass Ionomer	Powder: 95%strontium flouro-aluminosilicate glass and 5% polyacrylic acid Liquid: 40% aqueous polyacrylic acid	Equia Fil GC	GC Corporation, Tokyo, Japan

### Methods

#### Ethical approval

All procedures of this study adhered to ARRIVE guidelines. This in vivo experimental study was designed and conducted with the ethical approval number (19-248) of the National Research Centre (NRC) for conducting the experiments on animals. All methods were performed in accordance with the guidelines and regulation of Ethical Committee of the NRC.

#### Sample size calculation

A power analysis was designed to have adequate power to apply a statistical test of the null hypothesis that no difference would be found between tested groups regarding dentin bridge formation. By adopting an alpha (α) level of (0.05), a beta (β) level of (0.2) (i.e., power = 80%), and an effect size (ω) of (0.520) calculated based on the results of a previous study^16^; the minimal required sample size (n) was found to be (51) samples (i.e., 13 samples per group). Sample size calculation was performed using R statistical analysis software version 4.4.2 for Windows (R Core Team (2024). R: A Language and Environment for Statistical Computing. R Foundation for Statistical Computing, Vienna, Austria. https://www.R-project.org/.)

#### Study design and grouping

After obtaining the ethical approval number (19-248), Four male mongrel dogs aged 1–2 years and weighing 25–30 kg were used in this study. A total of 112 teeth were used and equally distributed into four main groups; two treatment groups and two control groups. Where the used capping materials in the two treatment groups were MTA Angelus and the experimental Calcium aluminate–Calcium silicate-based paste. While the control groups were either negative or positive control. Where intact teeth represented the negative control while the positive one represents the exposed and restored tooth with no capping material. All of these four groups were represented and randomly allocated in each dog using block randomization. Groups were randomly assigned to different block; where each block contain 7 teeth. In each dog twenty-eight teeth were prepared with seven teeth in each quadrant which represent a separate group. The included teeth were; two incisors, one canine, two premolars, and two molars. Every two dogs were considered as separate subgroup according to the observation periods; one month and three months, with a total of 14 teeth in each group.

Groups were classified according to the applied protocol as follow:

**Group I:** experimental Calcium aluminate–Calcium silicate-based pulp capping material.

**Group II:** Mineral Trioxide Aggregate (MTA Angelus, Londrina-PR-Brazil).

**Group III:** positive control group where no pulp capping material was applied.

**Group IV:** negative control group where intact teeth were selected**.**

#### Preparation of the experimental material

Calcium aluminate (CA) phase was synthesized laboratory by mixing ultrapure limestone (99.8% CaCo_3_) and Alumina (99.6% Al_2_O_2_) with 1:1 ratio^17^. This combination was calcined for two hours at 1000°c then, it was ignited for six hours at 1500 °C. By increasing temperature and time, CA, C1_2_ A_7_ and CA_2_ was added then X-ray diffraction was carried out to verify the identity of the prepared powder. Finally, the produced powder was ground in an agate mill to obtain a fine homogenous mixture in a submicron scale range from 100 nm to 1 µm^18^.

While preparation of tri calcium silicate (C_3_ S) powder was carried out by firing cubes of CaO: SiO_2_ with 3:1 molar ratio. Then it was added to the ultra-pure limestone- quartz (99.6% SiO_2_) and % 0.5 boric acid at high temperature up to 1000 °C for 2 h^19^. Finally, the produced mixture was milled in the submicron scale and remodeled then fired at 1450 °C for two hours with repetition of the firing process until the reaction was complete.

The final produced experimental material was assessed by X-ray diffraction (XRD) and Transmission Electron Microscopy (TEM) ((JEM-1230) at 100 kV) to verify the presence of free lime and the size of the produced powder. The mean setting time was (32.70 ± 0.75 min) which was significantly higher than MTA. The mean microhardness was (56.50 ± 7.41 VHN) while the solubility showed weight increase by (6.29 ± 3.05) and the percentage of change in dimensions was (0.64 ± 0.78).

#### Animal model selection and preparation

A total of four healthy male mongrel dogs with complete set of permanent dentition and average weight were selected for this study with approximately one and half year old. Dogs were purchased from the animal housing unit of Cairo university. Each dog was separately housed in a kennel and observed before the operation to exclude diseased dogs. Afterwards, the experiment was carried in the animal house of Cairo University.

##### General anesthesia and dog preparation

After fasting the dogs for 12 h, general anesthesia was administrated with the following protocol. Each dog was injected by atropine sulphate (Atropine Sulphate, ADWIA, Egypt) 0.05 mg/kg body weight and 1 mg/kg body weight Xylazine Hcl (Xyla-Ject, ADWIA Co, Egypt) subcutaneously. However intravenous anesthesia was introduced by using ketamine Hcl (Keiran, EIMC, Egypt) and it was maintained using 25 mg/kg body weight thiopental sodium (Thiopental Sodium, EIPICo, Egypt)^20^.

All teeth were chemico-mechanically cleaned with 0.2% Chlorhexidine Gluconate (Parodontax, GlaxoSmithKline, England)^21^.

##### Preparation of Class V cavity

Standard Class V cavities were prepared on the cervical 1/3 of the facial surface of upper and lower tooth^22,23^. A modified metal band was used with a centrally prepared window to standardize the size of the prepared cavities to be 3mm ± 0.5 and 2 mm ± 0.5; mesiodistally and occlussogingivally respectively. While the cavity depth was standardized to be 3 mm ± 1mm by mounting a plastic stopper in the used bur^20^. A large round bur (size 7, MANI, INC, Japan) driven by low speed micromotor was used to prepare four cavities, then Copious amount of water was used to rinse the prepared cavity^21^.

A sterile small round bur (size 0.5, MANI, INC, JP) was used to centrally expose the pulp. Homeostasis was achieved by placing a sterile cotton pellet lightly moistened with saline solution over the exposure site for nearly 1–2 min while applying gentle pressure^24^. Then all cavities were dried using a sterile cotton pellet^20^.

##### Application of the capping material

The capping materials were applied for each group as follow:

**Mineral Trioxide Aggregate (MTA Angelus):**

One sachet of MTA was mixed with distilled water on a sterile glass slab for 30 s until a homogeneous mix was achieved. The mix was applied on the exposure site immediately with a small excavator^20^.

**The experimental Calcium aluminate–Calcium silicate capping material:**

Mixing of Calcium aluminate–Calcium silicate based powder with a polycarboxylate acid on a sterile glass slab until a homogeneous mix was achieved. The mix was applied as a thin layer on the exposure site using a small excavator^20^.

Then hybrid self-cured Equia Fil Glass Ionomer (GC, Tokyo, Japan) restorative material was applied to seal the prepared the cavity^25^.

##### Observation period

Dogs were kept under observation to assess the presence or absence of any infection. They were kept on soft diet and receive a dose of a nonsteroidal anti-inflammatory drug (Rimadyl Carprofen, Zoetis, USA) to decrease pain postoperatively. Dogs were followed up and constantly evaluated during the entire periods of the experiment which were 1 month and 3 months^26^.

#### Euthanasia of dogs

Each dog was scarified by rapidly injection of anesthetic overdose using 20 ml of 5% thiopental sodium solution according to the American Veterinary Medical Association (AVMA) Guidelines for the Euthanasia of Animals. Where two dogs were sacrificed after each observation period. Both the maxilla and the mandible were surgically removed and sectioned into two halves using the manual saw^20^.

#### Histopathological analysis

For soft tissue fixation, each tooth was placed inside a separate container filled with 10% formalin (Gomhorya Co, Egypt) for one week and every 48 h the solution was changed^27^. Then decalcification of the hard tissues was done by using 20% Formic acid and 25% Sodium citrate for four months^28^. Then each tooth was individuality separated with a thin rim of bone around it to obtain separate blocks. Afterwards, each tooth block was embedded in paraffin wax to allow buccolingual sectioning of 6 um thickness slabs. Those slabs were stained with Hematoxylin–eosin then they were examined by light microscope for histopathological evaluation and assessment of reparative dentin formation^26,28^. Histopathological evaluation was performed in a blinded manner. All histopathological slides were anonymized and coded by an independent researcher before analysis, and the pathologist was blinded to group allocation and treatment details during the entire scoring process.

#### Outcomes evaluation

##### Inflammatory response and soft tissue evaluation

The degree of pulpal inflammation was evaluated and scored based on the criteria stated by Silva et al.^29^ as shown in Table 2.

**Table 2:** Scores and corresponding interpretations used for histologic evaluation.

0	No inflammatory cells
1	Mild: A few scattered inflammatory cells
2	Moderate: Moderate inflammatory cells infiltration
3	Severe: Heavy inflammatory cells infiltration or abscess formation

##### Dentine bridge formation

The degree of dentin bridge formation was evaluated and scored based on the criteria stated by Muruganandhan et al.^30^ and Nowicka et al.^31^ as shown in Table 3.

**Table 3:** Scores and corresponding interpretations used for dentin bridge formation.

1	Absence of hard tissue bridge
2	Little communication between pulp capping material and pulp
3	Complete

#### Statistical analysis

Ordinal data were presented as frequency and percentage values and were analyzed using the Kruskal–Wallis test followed by Dunn’s post hoc test. *P*-values were adjusted for multiple comparisons using the False Discovery rate (FDR) method. The significance level was set at *p* < 0.05 within all tests. Statistical analysis was performed with R statistical analysis software version 4.4.2 for Windows (R Core Team 2024). R: A Language and Environment for Statistical Computing. R Foundation for Statistical Computing, Vienna, Austria. https://www.R-project.org/.)

## Results

**Fig. 1:**
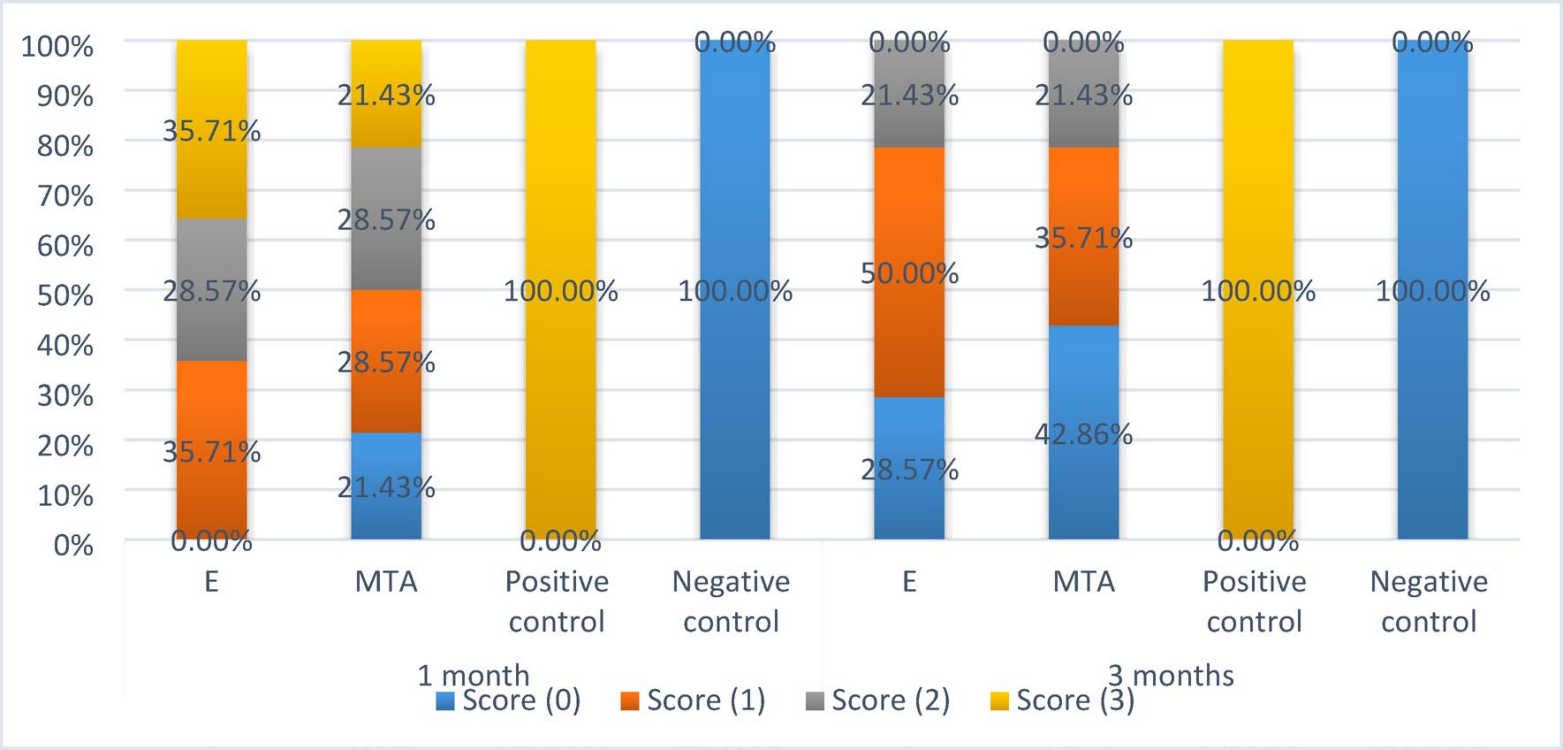
Stacked bar chart showing inflammatory response scores.

Summary statistics and the inter- and intragroup comparisons results for inflammatory response scores are presented in Table 4 and Fig. 1. Results showed that within both intervals, there was a significant difference between different tested and control groups.

**Table 4:** Statistical analysis of inflammatory response score.

Time	Inflammatory response score	n (%)				Test statistic	*p*-value
		Experimental Calcium aluminate	MTA	Positive control	Negative control		
1 Month	Score (0)	0 (0.00%)^A^	3 (21.43%)^B^	0 (0.00%)^A^	14 (100.00%)^C^	**40.22**	< 0.001*
	Score (1)	5 (35.71%)	4 (28.57%)	0 (0.00%)	0 (0.00%)		
	Score (2)	4 (28.57%)	4 (28.57%)	0 (0.00%)	0 (0.00%)		
	Score (3)	5 (35.71%)	3 (21.43%)	14 (100.00%)	0 (0.00%)		
3 Months	Score (0)	4 (28.57%)^B^	6 (42.86%)^B^	0 (0.00%)^A^	14 (100.00%)^B^	**41.92**	< 0.001*
	Score (1)	7 (50.00%)	5 (35.71%)	0 (0.00%)	0 (0.00%)		
	Score (2)	3 (21.43%)	3 (21.43%)	0 (0.00%)	0 (0.00%)		
	Score (3)	0 (0.00%)	0 (0.00%)	14 (100.00%)	0 (0.00%)		
Test statistic		**157.50**	**135.00**	**NA**	**NA**		
*p*-value		0.008*	**0.080**	**NA**	**NA**		

NA Not applicable. Values with **different superscripts** within the **same horizontal row** are significantly different, * significant (*p* < 0.05). Significant values are in bold.

After 1 month, there was a statistically significant difference between all groups at *p* value < 0.001. While, pairwise comparisons showed that there was statistically significant difference between MTA and the experimental Calcium aluminate group as the latter showed higher inflammatory scores with moderate/severe inflammation (64.3%) compared to MTA (50%).

After 3 months, also there was a statistically significant difference between all groups at *p* value < 0.001. While, pairwise comparisons showed no statistically significant difference between MTA and the experimental Calcium aluminate groups where, there was a reduction in the recorded inflammatory scores over time. MTA and the experimental Calcium aluminate groups showed low inflammatory scores with absence/mild inflammation in 78.57% of the samples in both groups.

**Fig. 2:**
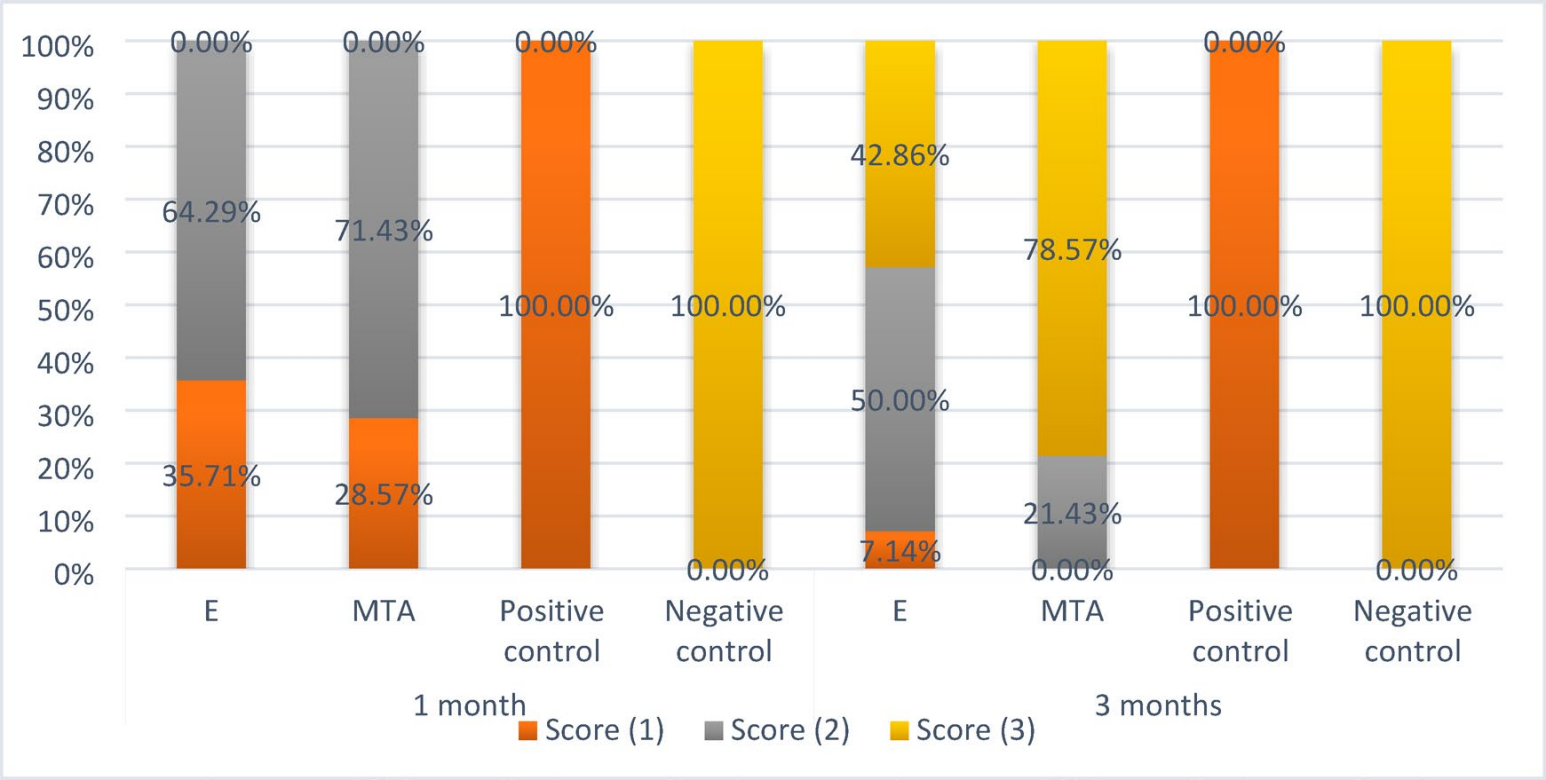
Stacked bar chart showing dentine bridge scores.

Summary statistics and the results of inter- and intragroup comparisons for dentine bridge scores are presented in Table 5 and Fig. 2. Results showed that within both intervals, there was a significant difference between different tested and control groups.

**Table 5:** Statistical analysis of dentine bridge score.

Time	Dentine bridge score	n (%)				Test statistic	*p*-value
		Experimental calcium aluminate	MTA	Positive control	Negative control		
1 Month	Score (1)	5 (35.71%)^B^	4 (28.57%)^B^	14 (100.00%)^B^	0 (0.00%)^A^	**43.51**	< 0.001*
	Score (2)	9 (64.29%)	10 (71.43%)	0 (0.00%)	0 (0.00%)		
	Score (3)	0 (0.00%)	0 (0.00%)	0 (0.00%)	14 (100.00%)		
3 Months	Score (1)	1 (7.14%)^A^	0 (0.00%)^A^	14 (100.00%)^B^	0 (0.00%)^A^	**41.95**	< 0.001*
	Score (2)	7 (50.00%)	3 (21.43%)	0 (0.00%)	0 (0.00%)		
	Score (3)	6 (42.86%)	11 (78.57%)	0 (0.00%)	14 (100.00%)		
Test statistic		**153.00**	**181.00**	**NA**	**NA**		
*p*-value		0.005*	< 0.001*	**NA**	**NA**		

NA Not applicable. Values with **different superscripts** within the **same horizontal row** are significantly different, * significant (*p* < 0.05). Significant values are in bold.

After 1 months, there was a statistically significant difference between all groups at *p* value < 0.001. While, pairwise comparisons showed no statistically significant difference between MTA, the experimental Calcium aluminate and the positive control groups. Both MTA and the experimental Calcium group showed partial dentin bridge formation in 71.4% and 64.2% of the samples respectively.

After 3 months, there was a statistically significant difference between all groups at *p* value < 0.001. While, pairwise comparisons showed no statistically significant difference between MTA, the experimental Calcium aluminate and the negative control groups. Both MTA and the experimental Calcium group showed either complete or partial dentin bridge formation in 100% and 92.8% of the samples respectively.

### Histopathological analysis

**Fig. 3:**
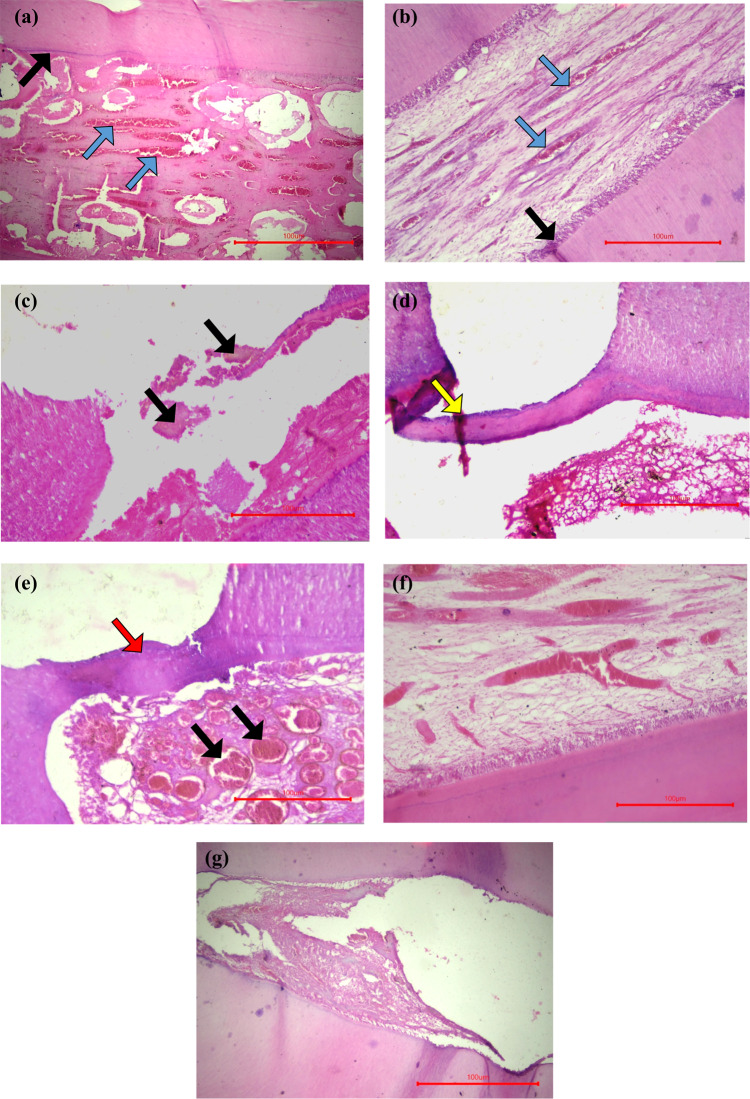
(**A**) of the experimental Calcium aluminate-Calcium silicate group after 1 month showing absence of odontoblastic layer (black arrow). Also note increased pulpal hyperemia (blue arrows). (**B**) of the MTA group after 1 month showing pulpal hyperemia (blue arrow) but with healthy intact odontoblastic layer (black arrow). (**C**) of the experimental Calcium aluminate-Calcium silicate group after 1 month showing calcific deposits at the exposure site (black arrow). (**D**) of the experimental Calcium aluminate-Calcium silicate group after 3 months showing dentin bridge attempt (yellow arrow). (**E**) of the MTA group after 3 months showing dentin bridge formation at the exposure site (red arrow). Note some pulpal hyperemia (black arrow). (**F**) of the negative control group showing healthy pulpal tissue, continuous predentin and intact odontoblastic layer. (**G**) of the positive control showing necrotic pulpal tissue with loss of all cellular details.

After 1 month, histopathological examination of the experimental Calcium aluminate group revealed varied inflammatory responses, ranging from hyperemia to disruption of the odontoblastic layer Fig. 3A. In contrast, the MTA group exhibited milder changes, with the majority of specimens showing only vasodilatation of the pulpal blood vessels Fig. 3B. Initial calcific deposits were observed at the exposure site in the experimental Calcium aluminate group Fig. 3C.

By 3 months, the experimental Calcium aluminate group demonstrated an attempt at dentin bridge formation Fig. 3D, while the MTA group showed a thicker and more uniform dentin bridge, accompanied by minimal tissue hyperemia and absence of inflammatory cells Fig. 3E.

The negative control group maintained intact pulpal morphology at both 1 and 3 months, characterized by healthy predentin, a continuous odontoblastic layer, and no signs of inflammation Fig. 3G. In contrast, the positive control group showed necrotic pulp tissue Fig. 3G.

## Discussion

Direct Pulp Capping (DPC) is a conservative approach aiming to preserve the tooth vitality and induce the formation of a hard tissue barrier over the pulp’s exposure site. Calcium silicate-based cements have been used as pulp capping materials to overcome the shortcomings of calcium hydroxide-based compounds.

According to various studies^32,33^ and a meta- analysis^34^, MTA consistently forms a better and more predictable calcific bridge than calcium hydroxide in direct pulp capping, with the privilege of having lower inflammation and reduced cytotoxicity^32^. However, its main disadvantage is prolonged setting time of approximately 3–4 h, poor mechanical properties and the potential for tooth discoloration, primarily due to the presence of bismuth oxide in its composition^35^. Therefore, Calcium aluminate cements have been introduced as pulp capping materials; as it exhibited desirable physical and chemical properties^36^, along with satisfactory marginal sealing ability and low toxicity^37^.

Calcium aluminate has been examined as an endodontic material in multiple in vitro and in vivo studies, demonstrating good antimicrobial action^38^ and no adverse effects, with outcomes comparable or even superior to those of MTA in subcutaneous implantation in rats^39^. In addition, Calcium aluminates produced a similar response to White Mineral Trioxide Aggregate (WMTA) in terms of inflammation after root canal filling in sheep teeth. Observations included newly mineralized apical tissue, thickening of the periodontal ligament and the ability to promote full bone recovery following bone defect repair^40^.

A study conducted by Nassar et al.^41^ proved that Calcium aluminate based experimental material showed good microhardness, dimensional stability and acceptable setting time. Moreover, the incorporation of C_2_S and C_3_S into the prepared experimental cement has a positive impact on bioactivity and mechanical properties. Added C_3_S/ C_2_S helps in proliferation and differentiation of human Dental Pulp stem cells (hDPSC) compared to C_3_S alone or Calcium hydroxide. This is proved by increased expression of odontogenic maker genes, strong alkaline phosphatase (ALP) activity and more mineralization process^42^. Compared to the frequently used materials, the composite materials proved to be biocompatible and raising the cell survival^43^.

In the same context, Andrei et al.^1^ concluded that the addition of Calcium silicate ions can induce odontoblasts’ differentiation and mineralization. On the other hand, polyacrylic acid was selected due to its known biocompatibility which in turns flavors the pulp repair procedure^44^.

Polyacrylic acid (PAA) can increase viscosity of liquid phase by means of the formation of intermolecular network structure^45^, and the presence of carboxyl groups on the PAA chains are essential as they bind with the released calcium ions (Ca^2+^) to form a three-dimensional (3D) network and cause gelation^46^.

In our study, dogs were selected to observe the effects of our smart innovative pulp capping material on vital pulp tissues, as it has been reported that similar reparative process occur among human and dog^47^.

In the present study, we evaluated the pulpal response to different pulp capping materials in dogs after 1 and 3 months. Similar study^48^ had employed the same 1-month observation period. A previous investigation confirmed the formation of osteodentin matrix within the first 2 weeks, followed by the development of a complete layer of reparative dentin at the capping site after 3 weeks^49^. Another study demonstrated the presence of a calcified bridge in all specimens 5 weeks after capping with MTA^50^.

At 8 weeks, Kang et al.^51^ compared the inflammatory response and calcific barrier formation induced by three Calcium silicate cements in a beagle pulpotomy study. The results showed reduced inflammation and thicker hard tissue formation at the exposure site compared to their previous in vivo study at 4 weeks^52,53^. However, findings in animal models may not always accurately reflect the responses in human teeth. For example, after 1 week of pulp capping in canine teeth, a complete hard tissue barrier was noted^54^, whereas in humans, the initiation of hard tissue formation has been reported to begin as early as 2 weeks after pulp capping^55^. Most studies revealed that in humans, a complete hard tissue barrier is typically formed between 30 to 42 days^56^. Moreover, Santos et al.^57^ concluded that that pre-operative pulp inflammation could strongly affects the prognosis of DP capping procedures. From the same perspective, carious-exposed pulp plays an important in clinical decisions regarding DPC where complete caries removal should be carried out to ensure good prognosis^58^.

After pinpoint exposure of intact teeth from dogs, the positive control group was restored entirely with glass ionomer to evaluate pulpal responses without any tested biomaterial.

Regarding the inflammatory response, our results revealed that the experimental Calcium aluminate–based pulp capping material induced a high level of pulpal inflammation after a 1-month follow-up period. Approximately 64% of the samples exhibited either moderate or severe inflammation, corresponding to scores 2 and 3. No statistically significant difference was observed between the experimental material and the positive control group; the latter as shown in Fig. 3G, however, showed severe inflammatory response (score 3) in 100% of the samples. In contrast, the MTA pulp capping material produced a comparatively lower degree of inflammation, as shown in Fig. 3B, with 50% of the samples exhibiting moderate to severe inflammatory response. Nevertheless, as reported in our results and shown in Fig. 3A and F, the MTA group still demonstrated significantly higher inflammatory scores compared to the negative control group. Initially higher degree of inflammation either in our experimental material or MTA PC could be due to the moderately long setting time. Healing pattern starts with an initial low-grade inflammation^59,60^. In the same context, Andrei et al.^1^ had stated that the initial inflammation could be an essential step for complete pulp healing provide that it should be limited and does not lead to severe necrosis. This is in agreement with Islam et al.^61^, a certain degree of pulpal inflammation following pulp capping is necessary for the healing processThe authors emphasized that during inflammation, the increase in pulp cell numbers, along with their proliferation and differentiation, plays a crucial role in inducing mineralized tissue formation at the exposure site. Moreover, Messias et al.^8^, had proved that Calcium aluminate material showed maintained cell viability through the initial phase of odontoblastic proliferation. On the contrary, Paula et al.^62^ observed that MTA induced a severe degree of inflammation after 3 days, which subsequently decreased to a mild-to-moderate level by the end of one month. Similarly, Kim et al.^63^ stated that MTA was associated with only mild pulpal inflammation after one month. In 2024, Janković et al.^10^, proved that their experimental Calcium aluminate–based pulp capping material resulted in the absence of pulpal inflammation in the majority of the tested samples.

On the other hand, after the 3 month follow up period, the experimental Calcium aluminate based pulp capping material successfully recorded reduction in the degree of pulpal inflammation, with only nearly 21% of the samples showing a moderate inflammatory response. The reduction was evident to the extent that no statistically significant difference was found between experimental material and MTA. Furthermore, both the experimental Calcium aluminate and MTA groups showed no significant difference compared to the negative control group, as all demonstrated a lower degree of inflammation when compared to the positive control group, which continued to exhibit severe inflammation (score 3) in 100% of the samples after 3 months.

These results are in accordance with Kavitha et al.^64^ who reported that, after 3 months, all samples in the MTA group maintained a healthy pulp with complete absence of inflammatory cells. Long-term studies on experimental Calcium aluminate–based capping materials are not yet available.

Regarding dentin bridge formation, as shown in Fig. 3C, our results revealed that the experimental Calcium aluminate–based pulp capping material induced the dental pulp to form a dentin bridge after a 1-month follow up period. Minimal communication between the capping material and the pulp tissue was observed in the Calcium aluminate and MTA groups, with 64.29% and 71.43% of the samples, respectively, showing dentin bridge formation. No statistically significant difference was noted between the two groups. In contrast, the positive control group failed to induce dentin bridge formation in 100% of the samples after 1 month.

It was proved that Ca and Si ions release can promote stem cells differentiation into odontoblast-like cells which in turns positively influence the repair process^65,66^ Several researches had proved that Calcium ions is among the constituents of both MTA and Calcium aluminate based experimental material. Where, Calcium ion release is essential for development and differentiation of hard tissue forming cells such as dental pulp cells and osteoblasts. This favors dentin mineralization and hard tissue barrier formation^67^; the released calcium ions interact with phosphate radicles/ions from the surrounding tissue fluids, leading to formation of apatite-like precipitation on the surface of MTA in situ. This process stimulates cell proliferation and decrease dentin permeability if deposited in dentinal tubules^67^. These findings are in agreement with Janković et al.^10^, who demonstrated that an experimental nanostructured Calcium aluminate pulp capping material promoted a higher percentage of dentin bridge formation after direct pulp capping. Moreover, Kim et al.^63^ and Okamoto et al.^68^ proved that MTA successfully induced the formation of dense and thick reparative dentin after a 1-month follow-up period. In the same context, it was proved that Calcium aluminate is capable to induce dentin bridge formation due to its chemical composition, as it releases calcium and hydroxyl ions necessary for precipitation of carbonated apatite and stimulation of hard tissue regeneration^69^.

On the other hand, after the 3-month follow-up period, the experimental Calcium aluminate–based pulp capping material successfully induced greater dentin bridge formation as shown in Fig. 3D. Among the samples, 42.86% exhibited complete bridge formation and 50% showed partial bridge formation. Similarly, as shown in Fig. 3E, the MTA group demonstrated 78.5% complete and 21.4% partial dentin bridge formation. No statistically significant difference was observed between these two groups and the negative control group, which showed 100% complete dentin bridge formation. On the contrary, the positive control group failed to induce reparative dentin formation in all samples.

These results are in agreement with Alnour et al.^70^ who reported that all samples in the MTA group exhibited complete dentin bridge formation after a 3-month follow-up period, and Mohanty et al.^71^ who concluded that MTA pulp capping material could promote both high quality and quantity of dentin bridge formation after 3 months. On the other hand, Silva et al.^34^ in their systematic review claimed that such studies should be interpreted cautiously as they couldn’t identically simulate the diverse clinical scenario.

The main limitation of the present study is that the evaluation of pulpal response was performed on healthy, intact teeth from dogs. Therefore, the results may not fully reflect the effects of newly developed experimental materials on inflamed pulps. Nevertheless, current advances in vital pulp therapy generate optimism for achieving natural tissue repair through the use of such smart bio active materials. This will help to shift towards the concept of regenerative endodontic therapy (RET) to attain a biological seal instead of the mechanical seal that could be achieved with artificial obturating materials^72^.

## Conclusion

Within the parameters of this study, it can be concluded that the new experimental Calcium aluminate–based biomaterial is a promising material for use in vital pulp therapy. It demonstrated similar inflammatory pulp response and dentin repair capability as MTA Angelus.

## Limitation

This study was carried along only three-month observation periods on intact teeth and healthy pulp tissues, with no previous pulp irritation. In addition, longer observation periods with large sample size might accurately reflect the long term durability of the tested materials.

## Recommendation

It is strongly recommended that further experimental and clinical studies be conducted to validate and confirm these outcomes in carious teeth with different degrees of pulpal inflammation with longer observation periods.

## Data Availability

The datasets used and/or analyzed during the current study are available from the corresponding author on reasonable request.
